# Use of Pressurized Air Infusion For Pre Descemet's Endothelial Keratoplasty (PDEK) - The Air Pump Assisted PDEK Technique

**DOI:** 10.2174/1874364101812010175

**Published:** 2018-07-23

**Authors:** Soosan Jacob

**Affiliations:** 1 Agarwal's Refractive and Cornea Foundation,Chennai,India; 2 Agarwal's Eye Hospital,Chennai,India

**Keywords:** PDEK, AC, Air pump, PI, Air infusion, DMEK

## Abstract

**Purpose:**

To assess the advantages offered by the air pump assisted PDEK technique that utilizes pressurized Anterior Chamber (AC) air infusion.

**Methods:**

Pressurized air infusion was provided through an anterior chamber maintainer connected to the fluid air exchange system of a posterior vitrectomy machine during surgery.

**Results:**

Pressurized air infusion within the AC helped perform Descemetorhexis, prevented bleeding during Peripheral Iridectomy (PI) and synechiolysis, prevented oozing of blood from peripheral corneal neovascularization into the AC and thus helped maintain a non-fibrinous AC environment. In addition, it helped in precise graft manipulation, centration, edge unfolding and unwrinkling after it was floated against the stroma as well as faster graft adhesion. It also prevented AC depth fluctuations during intra-cameral maneuvers and prevented intra-operative as well as post-operative graft detachment.

**Conclusions:**

This technique makes several steps of surgery easier and improves graft adhesion.

## 1 INTRODUCTION

As compared to Descemet's Membrane Endothelial Keratoplasty (DMEK) [1, 2] Pre Descemet's Endothelial Keratoplasty (PDEK) [3, 4] differs in providing the opportunity to use young donor corneas, thus potentially obtaining a larger quantum of cell transfer as well as younger and healthier cells. The splinting of the Descemet's Membrane (DM) and endothelium by the Pre Descemet's layer [5] gives the graft some rigidity. The Pre Descemet's layer also makes the PDEK graft resistant to tearing, thereby making it more easy to handle. However, unless appropriate techniques are followed, PDEK can still be challenging to perform and have a steep learning curve similar to DMEK [6-8].

In order to facilitate easier surgery, the author has described a technique (air-pump assisted PDEK) utilizing infusion of pressurized air into the AC. This facilitates graft handling within the Anterior Chamber (AC) and makes the procedure comparable in ease to DSAEK [9, 10] while giving advantages of DMEK in terms of thickness of graft and lack of stroma. It also helps prevent a variety of complications ranging from inability to unfold the graft to complex Descemet's detachments [11].

## 2 TECHNIQUE

Institutional review board approval was obtained and procedure confirmed to declaration of Helsinki. Informed consent was obtained from all patients. Data was analyzed using Microsoft Excel (Microsoft Corp, Redmond, Washington, US).

### 2.1 Procedure for Peparation of Donor Graft

The corneo-scleral graft was placed endothelium side up on a flat surface. A 30 gauge needle mounted on a 5 ml syringe half filled with air was bent bevel-up and inserted into the scleral part of the rim close to the limbus. It was then moved forward superficially such that the bevel of the needle lay completely within the peripheral part of the donor cornea. Air was then injected quickly in order to create a type 1 big bubble. If required, the bubble was further expanded carefully using either air, storage medium or 0.1% Trypan blue (Blurhex, Senses Pharma, India) after moving the needle into the bubble space. A 15 degree blade was used to enter the bubble at the base and Trypan blue was injected into the space to stain the graft. The graft was then cut all around its base with a curved Vannas scissor held flat to obtain a PDEK graft. The graft was stored again in storage medium till further use.

### 2.2 Procedure Followed on Patient’s Eye

**Fig. (1):**
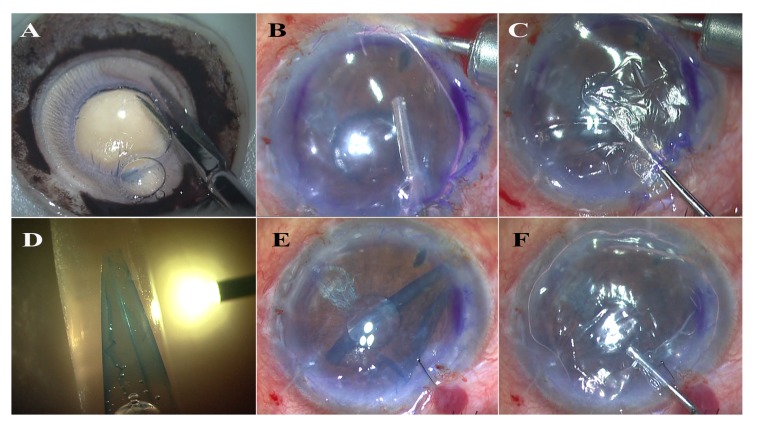
**A-** Type I bubble dissected to form PDEK graft; **B-** Anterior Chamber Maintainer (ACM) connected to air pump is fixed and inferior peripheral PI performed with vitrector. Notice absence of bleeding because of air tamponade; C**-** Descemetorhexis performed under pressurized air infusion; **D-** E-PDEK technique used to enhance visualization of PDEK graft; **E-**Air infusion is stopped and air replaced with BSS. The PDEK graft injected into AC seen under microscope light; **F-** Graft unfolded and air injected to float it up. Notice small edge folds on the graft.

**Fig. (2):**
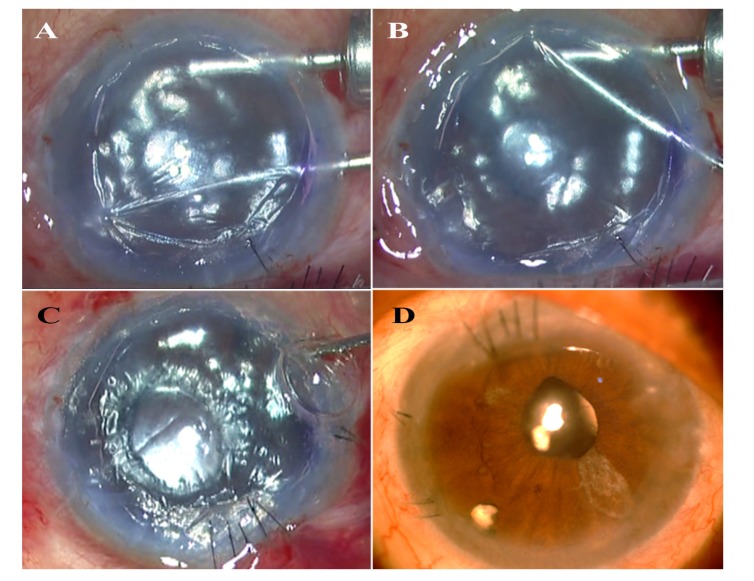
**A, B** The air pump is turned on again to get better air fill; The graft is centered into position and infolded peripheral graft edges are unfolded using a reverse Sinskey hook while the air acts as a third hand preventing the graft from dislocating; **C-** Any graft wrinkles are cleared. Continuing air infusion allows early graft adherence; **D-** 2 weeks post-operative appearance showing a clear graft.

A 1 mm paracentesis incision was created tangentially such that the Anterior Chamber Maintainer (ACM) could be placed directed towards the angle rather than to the centre of the AC. The fluid air exchange system in a posterior vitrectomy system was used to provide pressurized air infusion into the AC through the ACM. Because of continuous leakage of air, especially when any instruments were passed through the incisions, air pressure on the gauge of the machine was adjusted to obtain slightly higher than physiological IOP within the anterior chamber as checked by finger pressure. A partial entry, either clear corneal or scleral tunnel was created using a 2.8 mm bevel up keratome. Under air, an inverse Descemetorhexis was performed using a reverse Sinskey hook. In cases with extensive and long standing bullous keratopathy with thickened DM and largely non-functional endothelium, the entire host DM was stripped. In eyes with early bullous keratopathy, the area of DM stripped was planned to be 1 mm larger than the PDEK graft size. Gross edge irregularities and DM tags were smoothened out by localized Descemet's stripping with the reverse Sinskey hook or a microforceps. Under air, a small inferior Peripheral Iridectomy (PI) was made using the vitrector at 400 cuts per second and vacuum of 200 mmHg. Air was replaced with BSS once any iris bleeding stopped and a bubble test was performed to assess stability of the air bubble in the AC. In cases with iris defects or where the air bubble was unstable and tended to migrate posterior to the iris, the ACM was connected to balanced salt solution and a pupilloplasty was performed to attain a stable iris IOL diaphragm as described by Jacob et al in 2014 [12]. The ACM was then removed, the graft was loaded into a micro incision lens injector and after verifying correct graft orientation within the cartridge using the E-PDEK/ E-DMEK technique [11, 13], the graft was injected into the AC. Again, using the endoilluminator assisted visualization, the graft was unrolled and then floated up by injecting air with a cannula. The ACM was reinserted carefully posterior to the graft and continuous air infusion restarted. The pressurized air infusion allowed clear visualization of the graft against the stroma. Under pressure of air, the reverse Sinskey hook was used to hold the PDEK graft at a single point in the periphery and the graft was pulled to centration within the Descemetorhexis. The main port was then sutured. Any folded graft edges, if present, were then unfolded gently in one movement using a reverse Sinskey hook to hold the fold at its centre and gently pulling it open. Repeated and multiple points of graft touch were avoided. Graft wrinkles if present, were also stretched out using the reverse Sinskey hook, holding the graft only at its extreme periphery. During these maneuvers, the graft was prevented from dislocating by continuous support from pressurized air infusion. In case of excessive graft movements while unfolding graft edges, the graft was brought to the desired position and pressure allowed to rise temporarily in order to allow the graft to adhere to overlying stroma, thereby decreasing excessive mobility. Unfolding was then proceeded with and any other required intra-ocular manoeuvres were also performed. Additional sutures were applied to the main port if required to get an air-tight AC. The ACM was finally removed and the limbal paracentesis also sutured. After loosening the speculum, air and BSS were used to bring the eye to normal Intra-Ocular Pressure (IOP) and to obtain a 90% air fill. Light perception was checked and the patient asked to maintain a supine position (Figs. **1** and **2**).

## 3 RESULTS

12 patients were included in the study. Ten patients had pseudophakic bullous keratopathy, one patient had ICE syndrome, while one had viral endothelitis. All patients underwent underwent air pump assisted PDEK, 4 patients also required additional synechiolysis and three patients required pupilloplasty to reform the iris-IOL diaphgram. Continuous pressurized air infusion helped perform intra-operative steps and tamponaded any intra-ocular bleeding during PI and synechiolysis. Two patients had partial graft detachment. One was small and peripheral and reattached spontaneously while the other needed rebubbling on the slitlamp. All corneas were clear post-operatively. The patients were followed up for 6 months. Spectacle corrected distance visual acuity improved from 0.13±0.13 to 0.62±0.31 post-operatively. Pre-operative donor specular microscopy was 3033±74 cells/mm^2^ and at 6 months was 1916±309 cells/mm^2^ (Fig. **2D** and Table **1**).

## 4 DISCUSSION

Graft handling, folds and wrinkles in the floated graft, edge folds and decentred graft are major intra-operative challenges for both PDEK and DMEK as is graft detachment post-operatively. The use of continuous pressurized air infusion makes PDEK easier by helping perform various steps in a reproducible manner. It thus makes PDEK an easier surgery to adopt and more repeatable.

Descemetorhexis initiated under an air bubble [14-16] or continuous low pressure air infusion [15] allows the DM to be stripped against the overlying stroma making it easier and giving better visualization and control. Sizing and centration become easier and tags are less likely to form as well as better removed if formed. Continuous pressurized air infusion is used for further manoeuvres as well in our technique. Making a PI under air stops any iris bleeding. Synechiolysis if required is also done under air, preventing blood in the anterior chamber as well as preventing a fibrinous microenvironment which can make graft manipulation difficult as well as increase post-operative inflammation and interface debris. Viscoelastic usage that can potentially interfere with graft adhesion can also therefore be avoided completely for both PI as well as synechiolysis. Use of continuous air also prevents blood from seeping into the AC in cases with peripheral corneal neovascularization secondary to long standing corneal edema.

The air-pump assisted PDEK technique is more forgiving with respect to graft edge folds and decentration as the sturdy nature of the graft allows these to be adjusted once the graft is floated up against the stroma. The graft can therefore be floated earlier without having to ensure perfect centration or an edge-to-edge unfolded graft unlike in DMEK. A third hand effect is obtained that prevents AC depth fluctuations and the graft is prevented from detaching by the air pressure during various manoeuvres. Graft centration and unfolding of edge folds therefore becomes easier.^16^ This is especially important as young donor grafts can scroll tighter and increased manipulation may be required if attempting to unfold the graft completely before floatation, sometimes still resulting in unsuccessful edge-to-edge unscrolling. Increasing graft manipulation can result in increased cell loss. Air pump assisted PDEK is therefore helpful in allowing earlier flotation of graft followed by handling of graft edge folds, wrinkles and creases with a reverse Sinskey hook after floatation. Care should however be taken to avoid repeated, rough or large areas of graft touch with the reverse Sinskey hook which can increase endothelial cell loss. In our cases, we found graft edge unfolding easy to perform under pressurized air infusion once some graft adhesion had initiated and it was generally possible with single touch points, only at the extreme edges of the graft. Care should however be taken to ensure graft centration before graft adhesion initiates as this may become difficult later. Though similar manoeuvres may be performed in DMEK with very low infusion pressures, in our experience, DMEK grafts had a higher rate of graft wrinkling and graft edge tearing because of the thinner and more fragile nature of the graft. Hence this technique, if used, should be performed only with great caution in DMEK.

Pressurized air infusion also allows adequate operating space within the AC that allows easy insertion of instruments without causing damage to the graft endothelium. The set infusion pressure on the machine does not equate to the intra-cameral pressure achieved because of continuous leakage through the incisions. The infusion pressure on the machine is therefore set to achieve a slightly higher than physiological intra-cameral pressure estimated clinically by gently indenting the cornea. This slightly higher air pressure within the AC also allows earlier and better graft adherence

One of the disadvantages of this study was the absence of comparison with a control group where pressurized air infusion was not used. Future, randomized, comparative studies assessing long term endothelial cell loss between these two groups is therefore desirable. Further studies are also required on the duration of the pressure rise and the quantum of pressure rise required in different patient scenarios and correlating this with endothelial counts.

## CONCLUSION

To conclude, air pump assisted PDEK increases the ease, rapidity and success of surgery while decreasing complications and acting as a third hand within the AC to stabilize the graft for certain important manoeuvres. Addition of this newer technique will help the learning surgeon to perform the surgery with greater ease and confidence while also helping the established surgeon.

## Figures and Tables

**Table 1:** Pre- and post-operative endothelial count after air pump assisted Pre-Descemet’s Endothelial Keratoplasty.

–	**Pre-operative Donor Count**	**Post-operative Endothelial Count**
**1.**	3009	1890
**2.**	3115	2340
**3.**	3006	2264
**4.**	3115	1860
**5.**	2931	1765
**6.**	3077	1693
**7.**	3003	2198
**8.**	2976	1409
**9.**	3010	1782
**10.**	3012	1749
**11.**	2959	1654
**12.**	3185	2391
